# Medium-Term Outcomes of Digital Versus Conventional Home-Based Rehabilitation After Total Knee Arthroplasty: Prospective, Parallel-Group Feasibility Study

**DOI:** 10.2196/13111

**Published:** 2019-02-28

**Authors:** Fernando Dias Correia, André Nogueira, Ivo Magalhães, Joana Guimarães, Maria Moreira, Isabel Barradas, Maria Molinos, Laetitia Teixeira, José Tulha, Rosmaninho Seabra, Jorge Lains, Virgílio Bento

**Affiliations:** 1 SWORD Health Porto Portugal; 2 Neurology Department Hospital de Santo António Centro Hospitalar do Porto Porto Portugal; 3 Department of Population Studies Abel Salazar Institute of Biomedical Sciences Porto Portugal; 4 Centro de Investigação em Tecnologias e Serviços de Saúde (CINTESIS) Abel Salazar Institute of Biomedical Sciences University of Porto Porto Portugal; 5 Epidemiology Research Unit Instituto de Saúde Pública Universidade do Porto Porto Portugal; 6 Orthopaedics Department Hospital da Prelada - Dr. Domingos Braga da Cruz Porto Portugal; 7 Physical Rehabilitation Medicine Department Rovisco Pais Medical and Rehabilitation Centre Tocha Portugal; 8 Engineering Department University Institute of Maia - ISMAI Maia Portugal

**Keywords:** knee, TKA, home-based telerehabilitation, digital physiotherapist, artificial intelligence, eHealth

## Abstract

**Background:**

Physical rehabilitation is recommended after total knee arthroplasty (TKA). With the expected increase in TKA over the next few decades, it is important to find new ways of delivering cost-effective interventions. Technological interventions have been developed with this intent, but only preliminary evidence exists regarding their validity, with short follow-up times.

**Objective:**

This study aimed to present the follow-up results of a feasibility study comparing two different home-based programs after TKA: conventional face-to-face sessions and a digital intervention performed through the use of an artificial intelligence-powered biofeedback system under remote clinical monitoring.

**Methods:**

The digital intervention uses a motion tracker allowing 3D movement quantification, a mobile app and a Web portal. This study presents the results of the previous single-center, prospective, parallel-group, feasibility study including an 8-week active treatment stage and further assessments at 3 and 6 months post-TKA. Primary outcome was the Timed Up and Go score, and secondary outcomes were the Knee Osteoarthritis Outcome Scale (KOOS) score and knee range of motion.

**Results:**

A total of 59 patients completed the study (30 in the digital intervention group and 29 in the conventional rehabilitation group) and follow-up assessments. During the active treatment stage, patients in the digital intervention group demonstrated high engagement and satisfaction levels, with an 82% retention rate. Both groups attained clinically relevant improvements from baseline to 6 months post-TKA. At the end of the 8-week program, clinical outcomes were superior in the digital intervention group. At the 3- and 6-month assessments, the outcomes remained superior for the Timed Up and Go score (*P*<.001) and all KOOS subscale scores (at 3 months, *P*<.001 overall; at 6 months, KOOS Symptoms: *P*=.006, Pain: *P*=.002, Activities of Daily Living: *P*=.001, Sports: *P*=.003, and Quality of Life: *P*=.001). There was progressive convergence between both groups in terms of the knee range of motion, which remained higher for standing flexion in the digital intervention group than the conventional group at 6 months (*P*=.01). For the primary outcome, at 6 months, the median difference between groups was 4.87 seconds (95% CI 1.85-7.47), in favor of the digital intervention group.

**Conclusions:**

The present study demonstrates that this novel digital intervention for independent home-based rehabilitation after TKA is feasible, engaging, and capable of maximizing clinical outcomes in comparison to conventional rehabilitation in the short and medium term; in addition, this intervention is far less demanding in terms of human resources.

**Trial Registration:**

ClinicalTrials.gov NCT03047252; https://clinicaltrials.gov/ct2/show/NCT03047252

## Introduction

Total knee arthroplasty (TKA) is the third most commonly performed surgery in the United States, with over 700,000 procedures performed annually [1]. According to the Centers for Medicare & Medicaid Services, the average Medicare expenditure for surgery, hospitalization, and recovery after TKA ranges from US $16,500 to $33,000 [2]. As a consequence of population aging, the incidence of TKA is expected to increase, leading to an exponential growth in costs [3]. Reducing costs of care is thus a priority, with several initiatives already in place, such as the implementation of Bundled Payment options and the Comprehensive Care for Joint Replacement models [4,5]. These are examples of a broader trend favoring discharge from hospital to home, as opposed to more costly facility-based care [6].

Physical rehabilitation, the evidence-based [7] standard of care immediately following TKA, is being increasingly delivered to TKA recipients at home. Indeed, current evidence indicates that home-based care is a viable, more cost-effective alternative to conventional outpatient rehabilitation [8-12].

In the in-home setting, telerehabilitation, involving continuous monitoring from physical therapists, has shown to be very well accepted by patients [13,14], with results comparable to conventional outpatient physical therapy [13,15,16] or face-to-face home rehabilitation [17]. Besides reducing health costs, telerehabilitation enhances therapy uptake while allowing professionals to remotely adjust rehabilitation programs. In recent years, more advanced technological solutions have emerged, which further enhance patient’s autonomy and minimize real-time human supervision. These solutions incorporate biofeedback systems with the intent of increasing both patient performance and adherence [18].

Although there is preliminary evidence of the benefits of such technologies [18], they are generally poorly interactive, include complex machinery, and still show a low evidence level, with no long-term validation available yet [18]. Alternatively, smart portable biofeedback systems coupled with motion-tracking sensors are appealing sophisticated solutions that hold great promise in the upcoming age of artificial intelligence-guided therapies [19]. Promising as these may be, we found only one randomized controlled trial (n=142) testing an interactive telerehabilitation solution based on inertial motion trackers after TKA [16]; however, in that study, the intervention was too short (2 weeks) to draw definitive conclusions, and the outcomes were similar in both groups (system against conventional rehabilitation) [16].

In a previous study, we tested an artificial intelligence-powered digital system for home-based physical rehabilitation that uses inertial motion trackers in order to digitize patient motion and provide real-time feedback on performance through a mobile app. This system also includes a Web-based platform that allows the clinical team to monitor each patient’s progress and adapt the programs remotely, with the help of machine-learning algorithms. In this single-center, parallel-group, feasibility study (Trial registration: Clinicaltrial.gov NCT03047252; n=59), we compared the digital intervention to conventional face-to-face home-based rehabilitation after TKA, over an 8-week program, to test patient acceptance, engagement, and compliance and assess its clinical impact. The digital intervention was generally very well accepted, with high compliance and satisfaction levels, and the clinical outcomes were superior to those of the conventional rehabilitation group, in terms of change between the baseline and the end of the program [20]. In the present study, we assessed the medium-term results (3 and 6 months post-TKA) of both rehabilitation programs.

## Methods

A complete description of the methods can be found in the previously paper published by Correia et al [20]. An abridged version is presented here.

### Sample Size Estimation

Sample size estimation was performed considering the primary outcome measure Timed Up and Go (TUG) test score, based on the study by Mizner et al [21] (baseline TUG SD 2.4 seconds), where patients performed a rehabilitation protocol broadly comparable to the one used in the present study. A minimal clinically important difference (MCID) change of 2.27 seconds was considered, based on the study published by Yuksel et al [22]. Considering a power of 90%, a two-sided significance level of .05, and a dropout rate of 15%, 55 patients would be needed to detect a 2.27-second difference between the two groups. Given the wide variation in the SD of the TUG reported by different authors—from 0.5 seconds [23] to 6.3 seconds [16]—we decided to increase the sample size to 70 patients in order to account for a greater variation than the one reported by Mizner et al *.*

### Eligibility Criteria

All consecutive patients admitted to Hospital da Prelada, Porto, Portugal, for primary TKA, between December 19, 2016, and January 16, 2018, were screened for eligibility. Subjects were included if they were ≥18 years old and had clinical and imaging evidence of hip or knee osteoarthritis, indication for TKA according to the patient’s orthopedic surgeon, the ability to walk (unaided or with assistive device), and a caregiver available to assist the patient after surgery.

### Exclusion Criteria

The exclusion criteria were as follows: admitted for revision TKA; contralateral knee osteoarthritis severely limiting patient mobility and ability to comply with a rehabilitation program; aphasia, dementia, or psychiatric comorbidity interfering with communication or compliance to the rehabilitation process; respiratory, cardiac, metabolic, or other conditions incompatible with at least 30 minutes of light-to-moderate physical activity; major medical complications occurring after surgery, which prevented discharge of the patient within 10 days after the surgery; other medical or surgical complications that prevent the patient from complying with a rehabilitation program; and presence of blindness or illiteracy.

### Allocation

Patients were assessed preoperatively and subsequently scheduled for elective TKA. On discharge, patients were allocated to one of two groups, using patient address as criterion. Subjects residing in areas outside the administrative limits of the city of Oporto were allocated to the digital intervention group. Conversely, patients residing within the administrative limits of the city were allocated to the conventional rehabilitation group.

### Blinding

The nature of the study did not allow blinding of patients. Patient assessment was performed by one trained investigator (JT) who was blinded to the study groups. Statistical analysis was performed by a blinded statistician (LT).

### Intervention

Both groups received an 8-week rehabilitation program starting on the day after discharge (7-10 days after surgery). The conventional rehabilitation group received a home-based supervised program provided by a physiotherapist, 3 times a week, for 1 hour (total of 24 hours of active treatment time).

The digital intervention group received an initial onboarding visit from the assigned physical therapist, who trained the patient or caregiver to use the system and then performed a supervised session with the patient, ensuring that the patient was able to interact with the system independently or with assistance from a caregiver. From then onward, patients performed the rehabilitation program solely through the use of the biofeedback system, under remote monitoring from the physical therapist. Patients were asked to perform independent sessions at least 5 times per week with a minimum duration of 30 minutes (ideally, total of 20 hours of active treatment time), but were not excluded in case of lower intensity.

### Ethics Approval of Research

The study was approved by the National Data Protection Commission (authorization number 1476/2017) and the local ethics committee at Hospital da Prelada. The methods were conducted in accordance with the approved guidelines. All patients and caregivers were informed about the purpose and procedures of the study; they provided written informed consent before inclusion. All patient data were anonymized and linked to the patient by a unique study number that did not contain any personal identifiers.

### Outcome Assessments

In our previous report, outcomes were measured 4 weeks into the rehabilitation program and at the end of the rehabilitation program (week 8) [20]. For this study, patients were reassessed at 3 and 6 months postsurgery (± 10 work days) through face-to-face visits.

Several studies suggest that the outcomes should be measured not only in terms of range of motion (ROM) [24-27], but also using patient-reported outcomes and a performance-based test [28,29].

The primary outcome was the TUG score [30], which measures the time that a person takes to rise from a chair, walk 3 meters, turn around, walk back to the chair, and sit down. This test was chosen because it is simple and practical, has high interrater reliability [31], and has been demonstrated to predict both short- [32] and long-term [33] function following knee arthroplasty.

The secondary outcomes were patient-reported outcomes, measured by the Knee Osteoarthritis Outcome Scale (KOOS) and knee ROM in degrees. The KOOS scale [34] was validated by Alviar et al for patients undergoing TKA [35]. The KOOS consists of 5 subscales: (1) pain, (2) other symptoms, (3) function in daily living (activities of daily living [ADL]), (4) function in sport and recreation, and (5) knee-related quality of life (QoL). Standardized options were given (5 Likert boxes), and each question was assigned a score from 0 to 4. A normalized score (100 indicating no symptoms and 0 indicating extreme symptoms) was calculated for each subscale.

Regarding knee ROM, since the system used in this study was a validated medical device for joint angle measurement, with a reported root mean square error of 3.5º in comparison to standard goniometry in the technical file, knee ROM was measured automatically by the system. Active ROM was measured in the following movements: lying, sitting, standing knee flexion, and sitting knee extension. For each exercise, the patient was asked to perform three repetitions, and the best value of the three was recorded.

Individual patient data that underlie the results reported in this article were submitted as supplementary information (Multimedia Appendix 1), which can be accessed through the online version of this paper.

### Statistical Analysis

Outcome analysis was performed using a per-protocol analysis. The impact of the interventions on the primary and secondary outcomes was evaluated while considering the change between the baseline and 3 and 6 months. Differences between the two study groups were performed using the independent samples *t* test or Mann-Whitney *U* test. The 95% CIs were determined using Hodges-Lehman estimator. Since outcomes were measured at three different time points (baseline, 3 months, and 6 months), a repeated measures of analysis was performed using a 3 × 2 analysis of variance with group as an independent factor and time as a within-subject factor. When necessary, logarithm or square root transformations were performed to obtain normally distributed variables. In all analysis, a significance level of 0.05 was considered.

### System Technical Specifications

**Figure 1:**
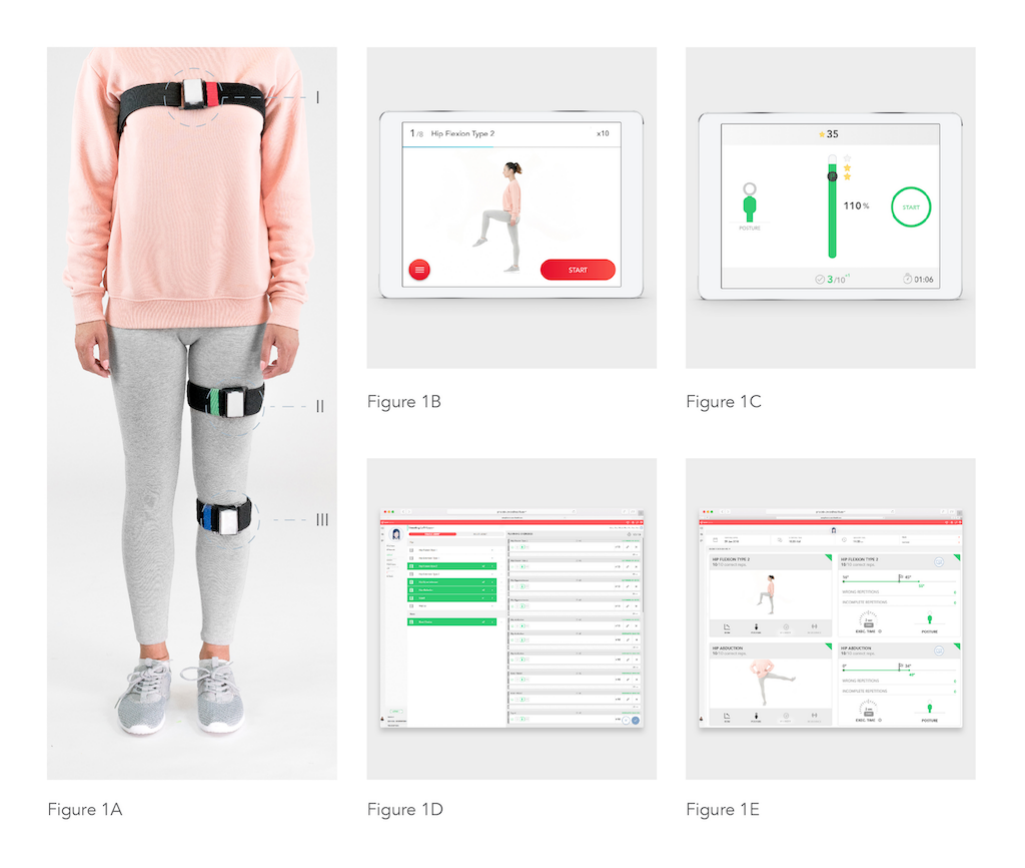
System components. (A) Motion tracker setup. (I) Red tracker: over the sternal manubrium. (II) Green tracker: anterior surface of the hip. (III) Blue tracker: over the anterior tibial crest. (B) Mobile App: preparation screen. This screen is shown before each exercise and displays a video of the exercise as well as audio instructions. (C) Mobile App: execution screen. (D) Web Portal - prescription screen. This screen displays the available exercises on the left and the layout of the exercise session on the right. (E) Web Portal - results screen. In this screen, the following information is presented: date and time of the session; session duration; pain and fatigue reported by the patient through the app; and one card per exercise, showing baseline and target joint angles, wrong and incomplete repetitions, and posture errors.

The system is composed of the following components (Figure 1).

#### Inertial Motion Trackers

Each tracker comprises gyroscopes, accelerometers, and magnetometers, allowing 3D movement quantification. The trackers communicate via Bluetooth low energy with a tablet computer. The trackers are placed on body segments using Velcro straps in specific positions.

#### Mobile App

Before each exercise, a video demonstration is presented to the patient (Figure 1) along with an audio explanation. During execution, the patient is given real-time visual and audio biofeedback through a dedicated interface (Figure 1). In each repetition, the patient is asked to fill a progress bar, earning a maximum of three stars if he/she surpasses the target range of motion. To do so, the patient must keep within prespecified movement and posture constraints (eg, excessive abduction in a straight leg raise is not allowed). If the patient performs a movement error or assumes an incorrect posture, an error message is displayed, with audio and video information on the specific error performed, thus allowing correction in the following attempts.

#### Web-Based Portal

The portal allows clinical teams to prescribe exercises, monitor results, and edit prescriptions (Figure 1).

## Results

**Figure 2:**
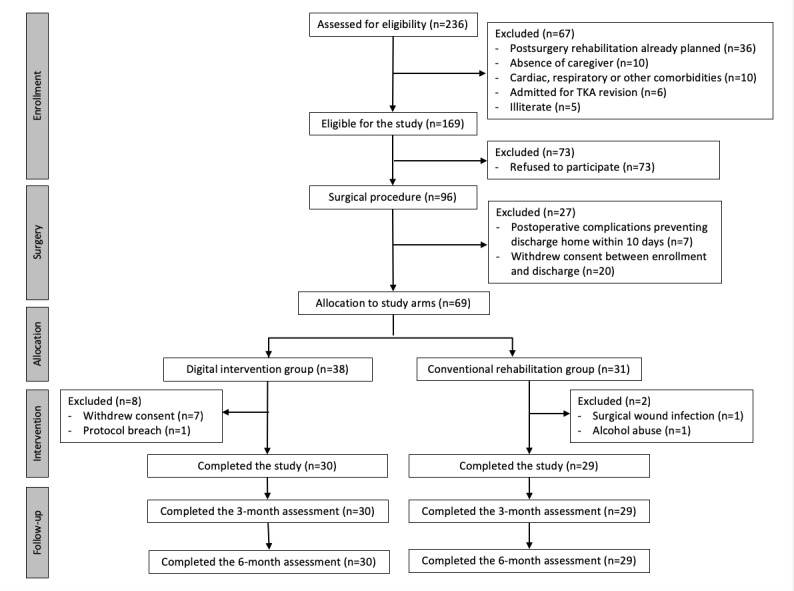
Study CONSORT diagram. CONSORT: Consolidated Standards of Reporting Trials; TKA: total knee arthroplasty.

In total, 59 patients completed the previous 8-week intervention study [20] (30 patients in the digital intervention group and 29 in the conventional rehabilitation group), and there was no loss to follow-up in this study. The CONSORT (Consolidated Standards of Reporting Trials) diagram is presented in Figure 2.

### Baseline Sample Characterization

Baseline characteristics of the study participants regarding demographics, comorbidities, and risk factors for adverse events as well as data on hospitalization and surgery are presented in Table 1. There were no differences between the two study groups regarding the abovementioned characteristics. In terms of primary and secondary outcomes, there were no differences between the two study groups regarding TUG and knee ROM (Tables 1 and 2). Regarding the KOOS, the digital intervention group had lower scores in every subscale [20] (Table 3).

**Table 1:** Baseline characteristics of the study participants.

Characteristics		Total (N=69)	Digital intervention group (N=38)	Conventional rehabilitation group (N=31)	*P* value^a^
**Demographics**					
	Age (years), mean (SD)	68.5 (7.0)	67.3 (6.8)	70.0 (7.2)	0.12^b^
	Gender, female, n (%)	54 (78.3)	32 (84.2)	22 (71.0)	0.30^c^
	Operated knee - right, n (%)	38 (55.1)	23 (63.2)	14 (45.2)	0.21^c^
**Comorbidities and known risk factors for adverse events**					
	Body mass index, mean (SD)	30.9 (4.9)	31.0 (4.5)	30.8 (5.4)	0.84^b^
	Smoking, n (%)	8 (11.6)	4 (10.5)	4 (12.9)	1.00^d^
	Hypertension, n (%)	48 (69.6)	25 (65.8)	23 (74.2)	0.62^c^
	Diabetes, n (%)	11 (15.9)	7 (18.4)	4 (12.9)	0.74^d^
	Pulmonary disease, n (%)	9 (13.0)	3 (7.9)	6 (19.4)	0.28^d^
	Cardiac disease, n (%)	4 (5.8)	2 (5.3)	2 (6.5)	1.00^d^
	Stroke, n (%)	0 (0)	0 (0)	0 (0)	N/A^e^
	Renal disease, n (%)	2 (1.4)	0 (0)	1 (3.2)	0.45^d^
	Bleeding disorders, n (%)	0 (0)	0 (0)	0 (0)	N/A
	ASA^f^ class 3 or 4^g^, n (%)	10 (14.5)	5 (13.2)	5 (16.1)	0.74^d^
	Steroids for chronic condition, n (%)	0 (0)	0 (0)	0 (0)	N/A
	Previous contralateral knee replacement, n (%)	17 (24.6)	7 (18.4)	10 (32.3)	0.30^c^
	Previous hip replacement, n (%)	3 (4.3)	3 (7.9)	0 (0)	0.25^d^
**Hospital admission and surgical procedure**					
	Time between admission and surgery (hours)	<24	<24	<24	N/A
	Operative time (min), mean (SD)	62.6 (11.3)	62.4 (9.87)	62.8 (13.0)	0.89^b^
	Minor adverse events before discharge, n (%)	1 (1.4)	0 (0)	1 (3.2)	0.45^d^
	Hospital length of stay (days), median (interquartile range)	6 (1.0)	6 (1.0)	6 (2.0)	0.83^c^

^a^Mann-Whitney *U* test*.*

^b^Independent samples *t* test.

^c^Chi square test.

^d^Fisher exact test.

^e^N/A: not applicable.

^f^ASA: American Society of Anesthesiology.

^g^American Society of Anesthesiology physical status classification system.

**Table 2:** Results of the secondary outcome measure (Knee Osteoarthritis Outcome Score).

Outcome variables		Digital intervention group, median (IQR^a^)	Control group, median (IQR)	*P* value^b^	Estimate difference between groups^c^	95% CI^c^
**Baseline**						
	Symptoms	34.0 (20.0)	50.0 (29.0)	<.001	–18.0	–25.0 to –17.0
	Pain	33.0 (12.0)	47.0 (24.0)	<.001	–11.0	–19.0 to –6.0
	ADL^d^	34.0 (18.0)	41.0 (18.0)	.005	–9.0	–15.0 to –3.0
	Sports	0.0 (0.0)	5.0 (8.0)	.006	0.0	–5.0 to 0
	Quality of life	13.0 (19.0)	25.0 (19.0)	.007	–12.0	–18.0 to 0
**At 3 months**						
	Symptoms	87.5 (11.8)	82.0 (19.5)	.01	9.0	0-15.0
	Pain	95.5 (11.8)	86.0 (22.5)	<.001	11.0	5.0-17.0
	ADL	93.0 (8.0)	87.0 (22.5)	.001	7.0	3.0-15.0
	Sports	30.0 (11.3)	20.0 (7.5)	.001	10.0	5.0-15.0
	Quality of life	81.0.0 (14.5)	56.0 (25.0)	<.001	19.0	12.0-25.0
**Change from baseline to 3 months**						
	Symptoms	51.5 (24.25)	25.0 (27.0)	<.001	25.0	15.0-35.0
	Pain	58.0 (12.0)	31.0 (23.5)	<.001	23.0	15.0-31.0
	ADL	57.5 (17.8)	35.0 (16.5)	<.001	20.0	13.0-27.0
	Sports	30.0 (11.3)	15.0 (10.0)	<.001	10.0	10.9-15.0
	Quality of life	65.0 (22.0)	44.0 (21.0)	<.001	25.0	18.0-37.0
**At 6 months**						
	Symptoms	96.0 (15.0)	86.0 (22.0)	.006	7.0	3.0-14.0
	Pain	100.0 (8.0)	86.0 (23.5)	.002	11.0	3.0-16.0
	ADL	97.0 (6.0)	87.0 (14.5)	.001	7.0	4.0-13.0
	Sports	42.5 (36.3)	20.0 (22.5)	.003	15.0	5.0-30.0
	Quality of life	94.0 (12.0)	63.0 (37.5)	.001	25.0	12.0-32.0
**Change from baseline to 6 months**						
	Symptoms	60.5 (25.8)	29.0 (33.5)	<.001	25.0	15.0-36.0
	Pain	61.0 (11.8)	39.0 (24.0)	<.001	20.0	14.0-28.0
	ADL	58.0 (17.5)	43.0 (23.0)	<.001	19.0	11.0-26.0
	Sports	40.0 (35.0)	15.0 (27.5)	<.001	20.0	10.0-30.0
	Quality of life	81.0 (20.0)	43.0 (40.5)	<.001	36.5	24.0-49.0

^a^IQR: interquartile range.

^b^Mann-Whitney *U* test.

^c^Hodges-Lehman estimator.

^d^ADL: activities of daily living.

**Table 3:** Results of the primary outcome measure (Timed Up and Go score).

Time point	Digital intervention group, median (IQR^a^)	Control group, median (IQR)	*P* value^b^	Estimated difference between groups^c^	95% CI^c^
Baseline	18.19 (6.2)	15.27 (8.5)	.13	2.02	–0.78 to 4.44
3 months	7.83 (2.4)	10.3 (3.5)	<.001	–2.50	–1.43 to –3.80
Change from baseline to 3 months	–10.28 (5.9)	–5.23 (8.5)	.004	–4.48	–1.64 to –7.37
6 months	6.86 (1.6)	8.74 (4.0)	<.001	–1.95	–1.24 to –2.90
Change from baseline to 6 months	–10.47 (7.2)	–5.08 (9.3)	.003	–4.87	–1.85 to –7.47

^a^IQR: interquartile range.

^b^Mann-Whitney *U* test.

^c^Hodges-Lehman estimator.

### Usability, Satisfaction, and Compliance Analysis in the Digital Intervention Group

Seven patients withdrew consent in the first week of the study, due to the inability to interact with the system. Of the remaining 30 patients, 18 (60%) required assistance of a caregiver for motion tracker placement or interacting with the app. There was no age difference between autonomous patients or those needing assistance (*P*=.19).

Only 4 patients (13%) did not comply with the recommended session frequency of 5 times per week.

Total active treatment time was superior in the digital intervention group (*P*=.005), with a median of 31.5 hours (interquartile range 18.0 hours; range 10.8-69.1 hours).

Patients had three face-to-face contacts with the therapist (one deployment session, one contact at 4 weeks, and one contact at the end of the 8-week program) and, on average, 0.4 (SD 0.7; range 0-2) additional face-to-face contacts as well as a median of 2.5 extra calls (interquartile range 3.0; range 1-12) for technical assistance.

Twenty-seven patients rated their satisfaction as 10/10, one with 9/10, and two with 8/10.

### Clinical Outcomes

The TUG scores were better (*P*<.001) in the digital intervention group (Table 3) in both 3- and 6-month assessments.

Concerning KOOS, the scores in the digital intervention group were higher than those in the conventional rehabilitation group for all subscales at both 3 and 6 months after TKA (Table 2).

Knee ROM was higher for sitting knee flexion (*P*=.046), sitting knee extension (*P*=.002), and standing knee flexion (*P*<.001) in the digital intervention group than in the conventional group at 3 months. At the 6-month assessment, only the standing knee flexion ROM remained significantly high (*P*=.01; Table 4).

#### Change Between Baseline and the 3- and 6-Month Assessments

At 3 months, the change in all outcome measures was superior in the digital intervention group and at the 6 months, this was true for the primary outcome (TUG), the KOOS score, and knee flexion while standing (Tables 2-4).

Based on the MCID reported in the literature for TUG (2.27 seconds) [22], clinically significant improvements were noted in both groups at 3 and 6 months, with participants taking 58% and 33% less time to complete the test in the digital intervention and control groups, respectively, at 6 months after surgery.

The difference between the median changes in the two groups was clinically significant, more than doubling the MCID (4.48 seconds at 3 months and 4.87 seconds at 6 months) in favor of the digital intervention group.

Regarding KOOS scores, the improvement noted in both groups was superior to the minimal important changes reported for the KOOS scores in subjects undergoing rehabilitation after TKA [36] (Symptoms: 10.7 points; Pain: 16.7 points; ADL: 18.4 points; Sports: 12.5 points; QoL: 15.6 points) in all subscales, denoting clinically relevant changes from baseline, 3 months, and 6 months after TKA (Table 2). The difference between the median changes in the two groups was also statistically and clinically significant in all subscales, again favoring the digital intervention group, except for the Sports subscale at the 3-month assessment, where the difference between the groups was lower than the minimal important change for this subscale (10.0 points; 95% CI 10.9-15.0).

**Table 4:** Results of the secondary outcome measures (knee range of motion).

Outcome variables		Digital intervention group, mean (SD)	Control group, mean (SD)	*P* value^a^	Estimate difference between groups	95% CI
**Baseline**						
	Lying flexion	80.7 (12.4)	84.7 (18.7)	.34	4.0	–12.2 to 4.3
	Sitting flexion	85.3 (16.0)	90.4 (13.1)	.19	5.1	–12.8 to 2.5
	Standing flexion	71.6 (20.3)	78.8 (16.6)	.15	7.2	–16.8 to 2.6
	Sitting extension	26.5 (8.4)	24.8 (7.8)	.43	1.7	–2.5 to 6.0
**At 3 months**						
	Lying flexion	100.1 (12.6)	93.3 (13.6)	.052	6.8	–0.04 to 13.62
	Sitting flexion	102.5 (13.1)	96 (11.3)	.046	6.5	0.10-12.89
	Standing flexion	95.6 (10.2)	84.9 (10.4)	<.001	10.7	5.22-16.08
	Sitting extension	11.8 (8.3)	19 (8.8)	.002	–7.2	2.73-11.65
**Change from baseline to 3 months**						
	Lying flexion	19.4 (15.5)	8.7 (15.1)	.009	10.7	2.8-18.7
	Sitting flexion	17.3 (20.1)	5.7 (14.7)	.01	11.6	2.4-20.8
	Standing flexion	23.9 (17.6)	6.1 (14.1)	<.001	17.8	9.5-26.2
	Sitting extension	–14.8 (9.0)	–5.9 (11.6)	.002	-8.9	–3.5 to –14.3
**At 6 months**						
	Lying flexion	103.4 (10.6)	101.5 (13.3)	.55	1.9	–4.38 to 8.15
	Sitting flexion	102.5 (10.8)	102.2 (12.3)	.93	0.3	–5.77 to 6.29
	Standing flexion	97.4 (9.9)	89.9 (11.7)	.01	7.5	1.78-13.08
	Sitting extension	7.1 (6.6)	9.7 (5.8)	.12	–2.6	–5.83 to 0.64
**Change from baseline to 6 months**						
	Lying flexion	22.7 (12.9)	16.8 (17.4)	.15	5.8	–2.1 to 13.8
	Sitting flexion	17.2 (19.1)	11.9 (13.9)	.22	5.4	–3.4 to 14.1
	Standing flexion	25.7 (20.1)	11.2 (14.0)	.002	14.6	5.5-23.6
	Sitting extension	–19.4 (8.4)	–15.1 (8.7)	.06	–4.3	–8.8 to 0.2

^a^Independent samples *t* test.

For knee ROM in patients undergoing TKA, there are no minimal important changes validated so far. The only comparable metric was reported in a study by Stratford and collaborators [37], which reported a minimal detectable change at a 90% CI of 9.6º for knee flexion and 6.3º for knee extension in patients after TKA. Hence, at 3 months, only the digital intervention group showed clinically relevant improvements in the knee ROM as compared to baseline assessment; however, this was true for both groups 6 months after TKA (Table 4). The difference in median changes revealed the superiority of the digital intervention over conventional rehabilitation at 3 months. At 6 months, only the mean change in the standing flexion knee ROM was significantly higher and clinically meaningful in the digital intervention group (14.6º; 95% CI: 5.5-23.6).

### Repeated Measures Analysis

This analysis was performed only for the normally distributed variables TUG and ROM after transformation. The results are summarized in Table 5.

**Figure 3:**
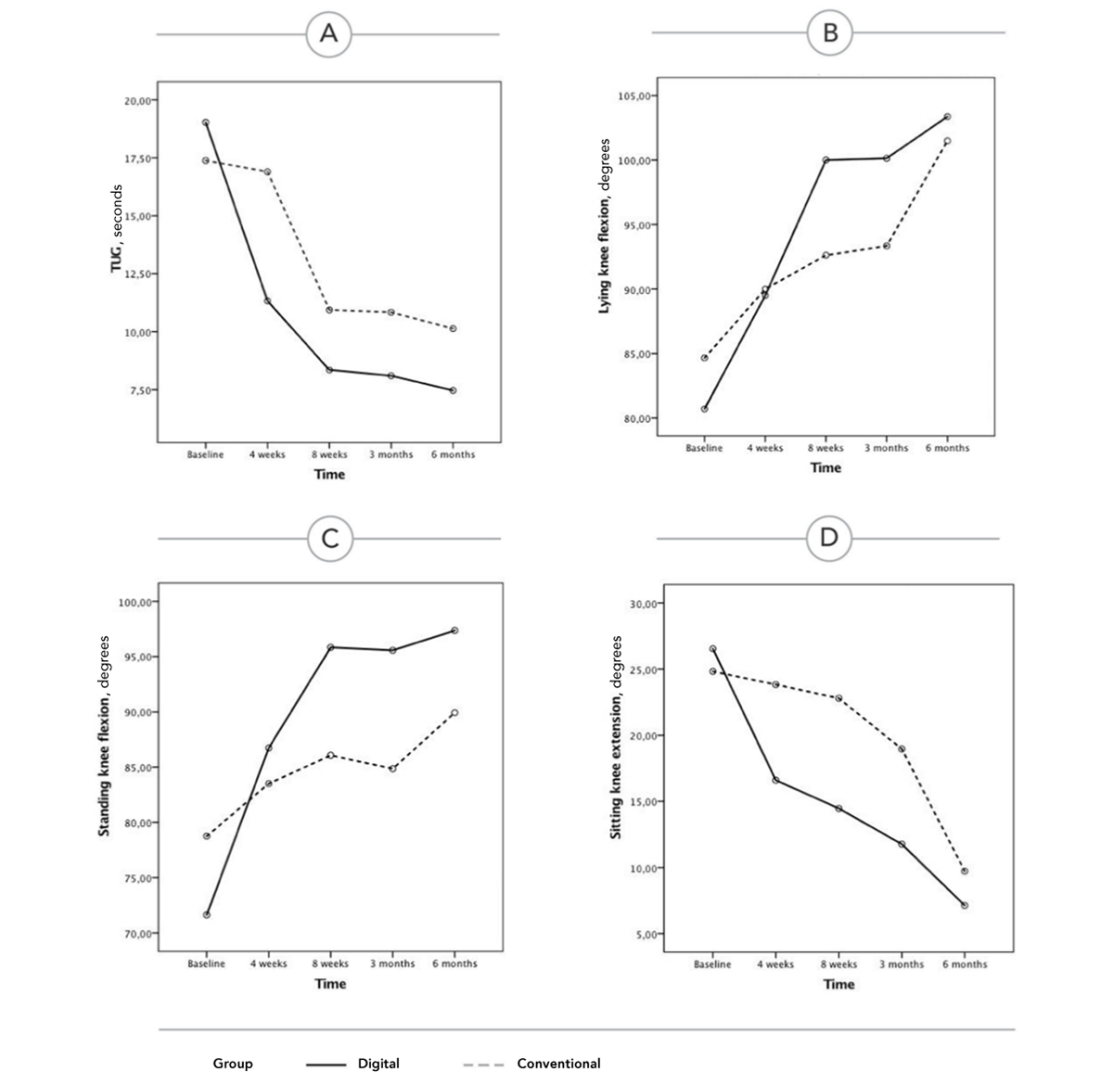
Evolution of the outcomes over time in both groups, based on the repeated measures analysis (estimated marginal means of transformed variables are presented). (A) Timed Up and Go score. (B) Lying knee flexion. (C) Standing knee flexion. (D) Sitting knee extension. TUG: Timed Up and Go.

For TUG, the repeated measures analysis revealed a main effect of time (*F*_2.2,124.5_=76.406, *P*<.001), a main effect of group (*F*_1,57_=9.346, *P*=.003), and an interaction between time and group (*F*_2.2,124.5_=7.807, *P*<.001) in favor of the digital intervention group (Table 5, Figure 3).

Regarding knee ROM, the repeated measures analysis revealed a main effect of time and an interaction between time and group in the four knee ROMs measured, again in favor of the digital intervention group (Table 5, Figure 3).

### Adverse Events

No adverse events were reported in any of the study groups in the period between the end of the active treatment stage and the 6-month assessment. In particular, there were no falls in any of the groups, readmissions to hospital for any reason, or TKA revision.

**Table 5:** Repeated measures analysis. Greenhouse-Geisser correction was used for all variables.

Outcome variables		Time			Group			Time*Group			
		*F*_df1,df2_	*F* value	*P* value	F_df1,df2_	*F* value	*P* value		*F*_df1,df2_	*F* value	*P* value
**Patient performance**											
	Timed Up and Go^a^	*F*_2.2,124.5_	76.406	<.001	*F*_1,57_	9.346	0.003		*F*_2.2,124.5_	7.801	<.001
**Knee range of motion**											
	Lying flexion	*F*_2.6,150.9_	42.3	<.001	*F*_1,57_	0.8	0.375		*F*_2.6,150.9_	4.29	0.008
	Sitting flexion	*F*_2.2,126.2_	24.8	<.001	*F*_1,57_	0.27	0.604		*F*_2.2,126.2_	3.98	0.02
	Sitting extension	*F*_3.0,169.4_	50.9	<.001	*F*_1,57_	11.4	0.001		*F*_3.2,169.4_	5.6	0.001
	Standing flexion	*F*_2.0,116.2_	37	<.001	*F*_1,57_	3.88	0.054		*F*_2.2,116.2_	9.17	<.001

^a^Logarithmic transformation.

## Discussion

The feasibility study was designed to assess both patient acceptance, engagement, and satisfaction with a novel digital intervention for rehabilitation after TKA and to estimate the clinical impact of the intervention in comparison to conventional face-to-face rehabilitation.

In terms of patient acceptance, the enrollment rate of this study was very low (29%), with patient refusal or consent withdrawal corresponding to more than half the screening failures. This was expected, given the relatively high mean age of the study participants (68.5 years; SD 7.0 years) and is a common issue in this field [16], likely representing patients’ skepticism toward new technological solutions as well as suspicion of possible hidden costs. This limitation can be overcome by ensuring better training and broader involvement of clinical teams (both doctors and nurses) that approach the patient upon admission.

From the patients initially allocated to the digital intervention, there was an 18% dropout rate in the first week, and 60% of the remaining patients needed assistance from a caregiver. Even if the number of additional face-to-face contacts for technical assistance was low, the number of extra calls for this reason was relatively high. This represents important usability issues faced by these new technologies in an older population and shows that there is room for improvement, namely, in facilitating tracker setup and removing physical interactions with the tablet. Nonetheless, in the patients who completed the 8-week program, user compliance with the program was very high, with only 4 patients using the system less than 5 days per week. Patient satisfaction was also very high. These are very promising results in terms of engagement, and they validate the gamification strategies in use.

Regarding clinical outcomes, the present study demonstrates clinically relevant improvements of all outcome measures in both groups at 3 and 6 months after TKA. We speculate that the good results obtained in both groups may be related to an early and intensive rehabilitation program.

When comparing the results obtained in the two groups, it is important to note that the study was sufficiently powered to detect clinically meaningful changes between the two groups, with posthoc analysis showing a statistical power of 95%.

Overall, this study demonstrates that the greater benefits observed in the digital intervention group for all outcome measures at the end of the 8-week assessment period were maintained at 3 and 6 months for the primary outcome (TUG) and KOOS score, with a convergence in terms of knee ROM (except for standing knee flexion). We speculate that maximizing short-term outcomes may also maximize medium-term (and possibly, long-term) outcomes. In addition, we speculate that one particular factor—patient empowerment regarding the rehabilitation journey—is maximized with an independent home-based program, possibly leading to a more active lifestyle and maintenance of some of the exercises included in the program. This may have, in turn, maximized the results. These aspects warrant further investigation in upcoming studies.

Regarding TUG, participants in the digital intervention group experienced a median change of 10.47 seconds (58% change from baseline) in the TUG test 6 months after surgery, while the control group experienced a median change of 5.08 seconds (33% change from baseline).

However, it must be noted that baseline TUG values in the present study were much higher than those reported by other authors, with preoperative values between 8 and 12 seconds, which in turn yield poor changes from baseline to the intervention time (approximately 8%-30% improvement) [21,38-40]. We could only find one randomized controlled trial (n=142) [16] with comparable baseline values for TUG (control: 22.8 seconds; SD 11.33 seconds and experimental: 18.9 seconds; SD 7.34 seconds). This study also compared an interactive virtual rehabilitation system for rehabilitation after TKA with conventional rehabilitation. However, in this study, the difference from baseline to 3 months was greater for the conventional rehabilitation group (10.86 seconds, SD 8.72 seconds; approximately 48% change) than for the digital intervention group (7 seconds, SD 6.31 seconds; approximately 37% change).

It is also important to note that the mean value reported for TUG at the 6-month follow-up assessment in the digital intervention group (6.9 seconds, SD 1.6 seconds) is similar to the value reported for healthy older individuals (50-85 years of age) by Bade et al (5.6 seconds, SD 1.0 seconds) and much lower than the value reported by the same authors for patients treated with conventional physiotherapy 6 months after TKA (9.1 seconds, SD 2.4 seconds) [41]. In the conventional group, the results at the 6-month assessment are in line with those reported by Bade et al [41].

Overall, the TUG analysis shows that important benefits were attained in both study groups; the results of the conventional group were in line with those reported by other authors, and those of the digital intervention group were superior to the results reported in the literature.

Concerning KOOS, Stevens-Lapsley et al [23] published a retrospective cohort evaluation on the self-reported and performance-based assessments of knee recovery following TKA. The scores obtained in this study for both groups surpassed those reported by these authors for KOOS subscales Symptoms, Pain, and ADL at all time points, but not for the KOOS subscale Sports. This could be explained by the fact that, in this study, baseline scores in the Sports subscale were much lower. Regarding the QoL subscale, the scores for the Sports subscale in the conventional rehabilitation group were slightly lower than those reported by Stevens-Lapsley et al [23] (3 months: 56.0 [SD 25] vs 63.3 [SD 2.98]; 6 months: 63.0 [SD 37.5] vs 66.96 [SD 3.01]), whereas the digital intervention group achieved much higher scores (3 months: 81 [SD 14.5]; 6 months: 94.0 [12.0]).

Overall, the results of the KOOS subscale scores demonstrate that for the comparison group, the clinical improvements were in line with those published by other authors, and results in the digital intervention group were much higher than those reported by other authors.

Regarding knee ROM outcomes, the results of knee flexion at 6 months in both groups were comparable to those reported in other studies (97º to 116º) [37], while active knee extension values were much lower than those found in the literature [37,41,42]. This latter difference could be a result of the more demanding position used to measure knee extension—sitting as compared to lying supine—which ultimately hampered direct comparison of the results.

Overall, differences between the intervention groups were not so evident, with results from all exercises converging at the 6-month assessment and entering a typical plateau phase, except for standing flexion, which showed higher amplitudes in the digital intervention group. However, importantly, short-term assessments (8 weeks and 3 months) revealed a much quicker improvement in the digital intervention group, potentially minimizing the time spent in rehabilitation after TKA surgery.

This study has several limitations that need to be acknowledged. First, it was a quasi-randomized study, where patient allocation was performed using a geographical criterion. Therefore, a number of factors (namely, socioeconomic) that were not controlled or addressed may have influenced the results. Nonetheless, both groups were similar in terms of baseline characteristics, except for KOOS scores, which were lower in the digital intervention group. It could be argued that the difference may be related to different health perceptions between the two groups, but the reason is not clear. Future studies should consider that pure randomization allows for a better control of these aspects.

Second, this was a single-center study performed in a low-volume orthopedic hospital, and all patients were admitted for elective surgery, which may not reflect the reality of other hospitals. In addition, the average length of stay (ie, 6 days) is higher than that reported in other studies [43], probably due to the inexistence of a fast-track protocol for TKA. The results reported here therefore need to be confirmed in multicentric trials in larger hospitals before generalization.

Third, the low inclusion rate may have represented a selection bias toward more technologically prone patients/caregivers, which needs to be properly addressed in future trials.

Fourth, treatment intensity was higher in the digital intervention group, which may have potentiated clinical results in this group. Nonetheless, even if this is the case, it is noteworthy that the superiority was maintained at the 3- and 6-month assessments.

Fifth, even though no serious adverse events were reported until the 6-month assessment, the absence of minor adverse events is more difficult to explain and was most likely due to an underreporting of these events. In future studies, besides direct telephone contact and specific questioning of adverse events in assessment appointments, event logs should be delivered to the patients to avoid underreporting.

In conclusion, the present study demonstrates that this novel digital intervention for rehabilitation after TKA is feasible and associated with high patient compliance and satisfaction. Like other novel technological approaches, it is still met with some skepticism by older patients, and usability still needs to be improved to ensure greater independence by users. This study also demonstrates that the digital intervention can maximize both short- and medium-term outcomes in comparison to conventional rehabilitation. As this approach is far less demanding in terms of human resources, this might be the first step toward a paradigm shift to artificial intelligence-assisted personalized electronic rehabilitation. These promising results warrant larger multicentric randomized controlled studies that address the study limitations to ensure widespread validation of this novel approach.

## Appendix

Multimedia Appendix 1 Raw data for outcome measures.

## Abbreviations

ASA: American Society of Anesthesiology
ADL: activities of daily living
CONSORT: Consolidated Standards of Reporting Trials
IQR: interquartile range
KOOS: Knee Osteoarthritis Outcome Scale
MCID: minimal clinically important difference
QoL: quality of life
ROM: range of motion
TKA: total knee arthroplasty
TUG: Timed Up and Go
